# Supplementary material for: Prediction of acoustic tinnitus suppression using resting-state EEG via explainable AI approach
Source: Sci Rep. 2025 Mar 31;15:10968. doi: 10.1038/s41598-025-95351-w (PMC11958676; doi:10.1038/s41598-025-95351-w)
Supplement: Supplementary file 1 — Supplementary Information. [file 41598_2025_95351_MOESM1_ESM.pdf]

Supplementary Material

Methods and supplemental information

EEG data and preprocessing

In the experiments performed at the University of Regensburg, resting state EEG was acquired for 10 minutes with alternating eyes open/closed blocks for 1 minute each on naive participants (i.e., no treatment or experimental procedures before the EEG recording). EEG was recorded with Brain Vision Recorder software (version 1.20, Brain Products GmbH, Gilching, Germany) together with a BrainAmp amplifier (Brain Products GmbH, Gilching, Germany) and an Easycap elastic electrode cap (Easycap GmbH, Herrsching, Germany) with 64 electrodes placed according to the 10-20 system[1,2]. EEG measurements were recorded at a sampling rate of 500 Hz and online referenced to FCz. Impedances were kept below 10 kOhm and balanced for all recordings.

In the experiments performed at the University of Zurich, the EEG resting state paradigm was identical to the Regensburg experiment and participants did also not undergo any treatment or experimental procedures before the EEG recording. The EEG system was from the same manufacturer but using a more current system with active electrodes. Brain Vision Recorder software (version 1.33, Brain Products GmbH, Gilching, Germany) in conjunction with a BrainAmp DC amplifier (Brain Products GmbH, Gilching, Germany) was used. Acticap Slim active electrode cap (Easycap GmbH, Herrsching, Germany) featuring 64 electrodes arranged in accordance with the international 10-20 system. Recordings were performed at a sampling rate of 1000 Hz, and online referencing was set relative to the FCz electrode. Impedances were kept below 10 kOhm and balanced for all recordings.

The EEG data was down-sampled to a sampling frequency of 250 Hz and before that, it underwent a lowpass filtering process with a cutoff frequency of 500 Hz to prevent signal aliasing in the down-sampling. Subsequently, the signals were filtered using a linear phase FIR filter with low and high cutoff frequencies of 1 and 80 Hz, respectively, in order to preserve the frequency content within different frequency bands. Additionally, a zero-phase FIR notch filter with a notch frequency at 50 Hz with stop and transition bands set to 0.25 and 1 Hz was applied to the data. This notch filter effectively eliminated any electrical interference or (line) noise around the 50 Hz frequency. The standard equidistant 10-20 system montage was used for electrode locations[1,2]. Using independent component analysis (ICA), the EEG data was separated into different components, and components related to eye blinks and eye movements were automatically identified and removed using the `MNE-ICALabel` python package[3]. The initial 3 seconds of each block (whether eyes open or closed) were excluded from the data to eliminate artifacts caused by the transition between eye opening or closing. The remainder of the EEG data blocks are then concatenated and segmented into 2-second epochs. We opted to use eyes closed epochs of the recordings to predict BATS to reduce vision artefacts and in accordance with previous works[4–6].

Feature extraction

In this study, we employed the `MNE` v.1.5.1 Python package[7] for preprocessing eeg recordings and computing power spectral density in both sensor and source spaces. The `FOOOF` v.1.1.0 Python package[8] was utilized to compute the aperiodic components (offset and slope) of the power spectral density. For assessing spectral connectivity within brain labels, we utilized the `MNE-Connectivity` v.0.5 package[9]. In addition, `scikit-learn` v.1.3.1[10] was used for the classification tasks, and the `SHAP` v.0.43.0 package[11] was employed to evaluate the direction and importance of features in the classification task.

Spectral band power

The average power of a signal was calculated within five specific frequency ranges: delta (0.5–4.5 Hz), theta (4.5–8.5 Hz), alpha (8.5–13.5 Hz), beta (15–30 Hz), and gamma (30–80 Hz)[12]. This computation was performed for each epoch using *multitaper* spectral estimation method[7,13], in which the signal is convolved with a set of optimal bandpass filters known as DPSS filters and the the final power spectral density is obtained by averaging resulting power spectra over all the filters. The bandwidth of the chosen tapers is set to 8 divided by the duration of each epoch, which is equivalent to 4 Hz. For a selected frequency range, the frequencies within ± half of the bandwidth (i.e., bandwidth / 2) are smoothed together to obtain the power estimate[14]. As a result, the number of band power features is equal to 310 (5 * 62), which represents the number of frequency ranges multiplied by the number of channels.

Spectral entropy

We computed Shannon entropy to quantify the degree of disorder or uncertainty in the distribution of power across different frequency components in a signal's spectrum. It provides a measure of the diversity or randomness of frequency content within a signal's spectral profile. Spectral Shannon entropy is calculated as follows for each individual epoch within each frequency range: first we computed the power spectral density (PSD) of the epochs within the specific frequency range as described in . Then we normalized the PSD values to obtain a probability distribution. This involves dividing the PSD values by the sum

of PSD values within that frequency range for that specific channel, ensuring that the probabilities sum up to 1. Finally we Calculated the Shannon entropy for that channel and frequency range using the following formula:

$$H = -\sum_{i=1}^{N} P(f_i) \cdot \log_2[P(f_i)] \tag{1}$$

where H represents the Spectral Shannon entropy. N is the total number of frequency components or bins in the spectrum and $P(f_i)$ is the probability (or normalized power) associated with the i th frequency component in the spectrum.

Aperiodic spectral power

In order to calculate the aperiodic broadband and exponent parameters for each epoch, we used the `FOOOF` Python module[8]. This package allows for the parameterization of the estimated power spectrum density (PSD). Specifically, it models the non-oscillatory portion of the PSD, which exhibits a 1/f-like behavior, using an exponential function as noted in the following equation:

$$AP(f) = 10^b \cdot \frac{1}{(k + f^x)} \tag{2}$$

where b is the broadband offset, k indicates the 'knee' parameter for controlling the bend in the spectrum and x denotes the aperiodic slope. By fitting this model, the aperiodic broadband offset and exponent parameters can be obtained for further analysis and interpretation. 124 (62 * 2) features representative of aperiodic activity used for classification purposes.

Source space power spectral density

To calculate an approximate forward operator for our EEG recordings, we utilized the boundary element model, source model, and co-registration information of a standard template MRI subject from the `MNE` python package[7]. In our EEG recordings, since there was no specific period of the data available for estimating the noise covariance, we used an ad hoc covariance matrix for noise modeling within our EEG sensors. This ad hoc covariance matrix is equivalent to what we would obtain for Gaussian noise on the sensors, assuming an infinite number of samples. This approach is a practical way to account for sensor noise in the absence of direct noise estimation from the data. Utilizing the noise covariance and forward solution, we employed the linear minimum-norm inverse method known as "*dSPM*" to determine the inverse solution[7]. This allowed us to obtain source time courses and source power spectra. For each brain label, we extracted a single time course by averaging across vertices at each time point within each label[7]. The brain labels and cortical parcellation were derived from the Desikan-Killiany Atlas[15]. The number of features that represent the power of brain labels is 340 which is equal to number of frequency ranges * number of brain labels.

Connectivity measure

To estimate spectral densities for coherence calculation, we employed a continuous wavelet transform utilizing Morlet wavelets with 7 cycles and a zero mean. The temporal window decreases proportionally with frequency, scaling by the number of cycles. The formula employed to subsequently compute coherence between two epochs at a specific frequency component is as follows:

$$C(f) = \frac{|S_{xy}(f)|^2}{S_{xx}(f) \cdot S_{yy}(f)} \tag{3}$$

where Cross-Power Spectral Density $S_{xy}(f)$ indicate the degree to which the two epochs are correlated at a specific frequency. It accounts for both phase and magnitude information. A high magnitude of $S_{xy}(f)$ suggests strong correlation. Power Spectral Densities $S_{xx}(f)$ and $S_{yy}(f)$ measure the power or energy contained in each epoch at the same frequency and the coherence value $C(f)$ indicates the relationship or coherence between the two epochs at frequency f ranging between 0 and 1. We computed the spectral connectivity between all pairs of brain regions delineated by the 'Desikan-Killiany' cortical atlas, considering the two distinct frequency ranges: alpha and gamma.

Feature importance and directionality

We used Gini index[16] as an indicator of feature importance in our classification process using RF with 100 trees (estimators) in the forest. In a classification problem, the Gini impurity for a node is calculated as follows:

$$Gini(node) = 1 - \sum_{i=1}^{C} (p_i)^2 \tag{4}$$

where C is the number of classes and p_i is the probability of an element in the node belonging to class i. Feature importance is determined by aggregating the decrease in Gini impurity achieved by each feature when it is used to split nodes across all the trees in the forest. Features that consistently lead to a significant reduction in Gini impurity are considered more important, as they contribute more to the classification accuracy.

To assess the directionality of the features on the model prediction, we used `SHAP` Python pacckage[11]. `SHAP` calculates Shapley values from cooperative game theory. Shapley values allocate a contribution score to each feature based on its collaboration with other features in predicting a specific outcome. These scores represent the impact of each feature on a model's prediction. Following is the equation to compute Shapley values for each individual feature:

$$\phi_i(f) = \frac{1}{N} \sum_{S \subseteq N \setminus \{i\}} \binom{N-1}{|S|}^{-1} (val(S \cup \{i\}) - val(S)) \tag{5}$$

in which, represents $\phi_i(f)$ the SHAP value for feature i. N is the total number of features. S is a subset of features that excludes feature i. $\mathrm{val}(S)$ represents the model's output (e.g., prediction) for the subset of features S and $\binom{N-1}{|S|}$ denotes the number of ways to choose a subset of features S from the remaining $N-1$ features. `SHAP` generates interpretable explanations for individual predictions (i. e. +BATS and -BATS). For a given prediction, it quantifies how much each feature contributed to pushing the model's prediction away from a baseline or reference prediction. This attribution helps to understand not only which features are important but also in which direction they influence the prediction. Positive SHAP values indicate that a feature contributes to increasing the prediction, while negative values suggest that a feature contributes to decreasing the prediction. This directionality allows to grasp whether a feature has a positive or negative impact on the classifier outcome.

Tables

Table S1. Sample description. All dB values are in dB SPL (sound pressure level). M = mean; SD = standard deviation; Md = median; BATS = brief acoustic tinnitus suppression. BATS levels refer to the perceived tinnitus loudness after sound stimulus offset.

	**Main dataset**					**Validation dataset**				
**N (female)**	73 (38)					29 (4)				
**Tinnitus side (left/ right/ bilateral)**	(7/ 13/ 53)									
	**M**	**SD**	**Md**	**Min**	**Max**	**M**	**SD**	**Md**	**Min**	**Max**
**Age (years)**	52.60	10.84	55.00	23.00	69.00	41.19	13.62	35.00	22.00	66.00
**Tinnitus duration (months)**	118.81	70.84	110.00	18.00	280.00	112.32	107.00	60.00	6.00	380.00
**Hearing loss (both ears, dB)**	21.70	12.41	21.69	-7.22	53.06	6.996	6.41	8.42	-1.91	18.44
**MML (dB)**	62.91	16.45	60.65	30.10	90.00	51.81	20.13	55.50	1.00	85.00
**THI total score (0-100)**	37.26	24.08	34.00	4.00	98.00	38.54	17.59	36.00	12.00	84.00
**GUF total score**	11.30	6.95	10.00	0.00	34.00	6.65	4.85	6.50	0.00	16.00
**Tinnitus loudness (dB)**	54.96	17.52	52.00	27.00	90.00					
**BATS (%) / BATS (-5 - +2)***	86.35	18.82	93.21	7.59	107.14	-0.92	2.23	0.00	-5.00	1.00

Table S2. Descriptive statistics of sample split in the main dataset. No differences in key variables between the groups except for MML (higher in -BATS). All dB values are in dB SPL (sound pressure level). SD = Standard Deviation.

Variable	Mean (SD)		Difference statistics	
	+BATS	-BATS	Statistics	p-value
Age (years)	54.40 (9.40)	51.24 (11.87)	t=1.255	0.214
Sex (female)	35 (16)	38 (22)	chi=0.650	0.420
THI total score (0-100)	34.86 (25.73)	39.47 (22.58)	t=-0.816	0.417
Hearing loss (both ears, dB)	25.38 (9.51)	23.06 (11.96)	t=0.914	0.364
tinnitus duration (months)	114.17 (73.32)	123.08 (69.17)	t=-0.534	0.595
tinnitus frequency	6098.14 (2684.83)	6122.39 (3229.57)	t=-0.035	0.972
MML (dB)	60.03 (16.87)	67.66 (14.84)	t=-2.054	**0.044**
tinnitus loudness (dB)	54.71 (22.46)	63.29 (22.03)	t=-1.646	0.104
Hyperacusis score (0-45)	10.34 (7.49)	12.18 (6.38)	t=-1.133	0.261

Table S3. Overview of brain regions extracted from Desikan-Killiany atlas and assigned to 9 sub-networks. For each brain parcel both left and right hemispheres are assigned to the correponding sub-network.

**Network name**	**Included brain labels**
**VSN** (visual network)	cuneus - lingual - lateraloccipital -pericalcarine
**SMN** (somatomotor network)	caudalmiddlefrontal - postcentral - precentral - paracentral - transversetemporal
**AUN** (auditory network)	superiorparietal - superiortemporal - transversetemporal
**VAN** (ventral attention network)	fusiform - inferiorparietal - lingual - lateraloccipital
**DAN** (dorsal attention network)	caudalmiddlefrontal - lateraloccipital - paracentral - superiorparietal - superiortemporal
**FPN** (frontoparietal network)	lateralorbitofrontal - parsopercularis - parsorbitalis - parstriangularis - rostralmiddlefrontal - superiorfrontal - superiortemporal
**DMN** (default mode network)	caudalanteriorcingulate - entorhinal - frontalpole - isthmuscingulate - medialorbitofrontal - parahippocampal - posteriorcingulate - precuneus - rostralanteriorcingulate
**DGN** (deep grey matter network)	caudalmiddlefrontal - paracentral - postcentral - precentral - superiorparietal - superiortemporal
**LBN** (limbic network)	caudalanteriorcingulate - entorhinal - frontalpole - isthmuscingulate - medialorbitofrontal - parahippocampal - posteriorcingulate

Table S4. Random Forest classifier performance. Metrics evaluating performance, such as accuracy, precision, recall, and F1-score, are provided for each classification task, encompassing both the primary dataset and the validation dataset.

	Main dataset				Validation dataset			
	accuracy	*precision*	*recall*	*f1-score*	*accuracy*	*precision*	*recall*	*f1-score*
**Sensor space features**	98%	98%	98%	98%	96%	96%	96%	96%
**Source space features**	98%	98%	98%	98%	99%	99%	99%	99%
**Connectivity features**	86%	86%	86%	86%	82%	82%	81%	80%

Figures

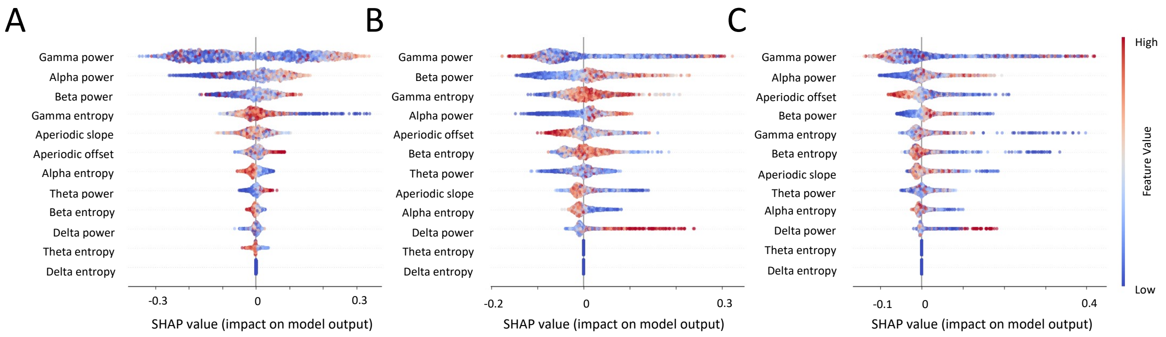

Figure S1. Importance order and directionality of sensor space features are shown for three different loudness threshold value, namely 90 (A), 70 (B) and 50 (C). The dots at each plot indicate SHAP values measuring how much each feature category contributes to predicting class of individuals with +BATS. Furthermore, the color of each data point (epoch) represents the feature values, following a gradient from red to blue, where red indicates high values, and blue signifies low values.

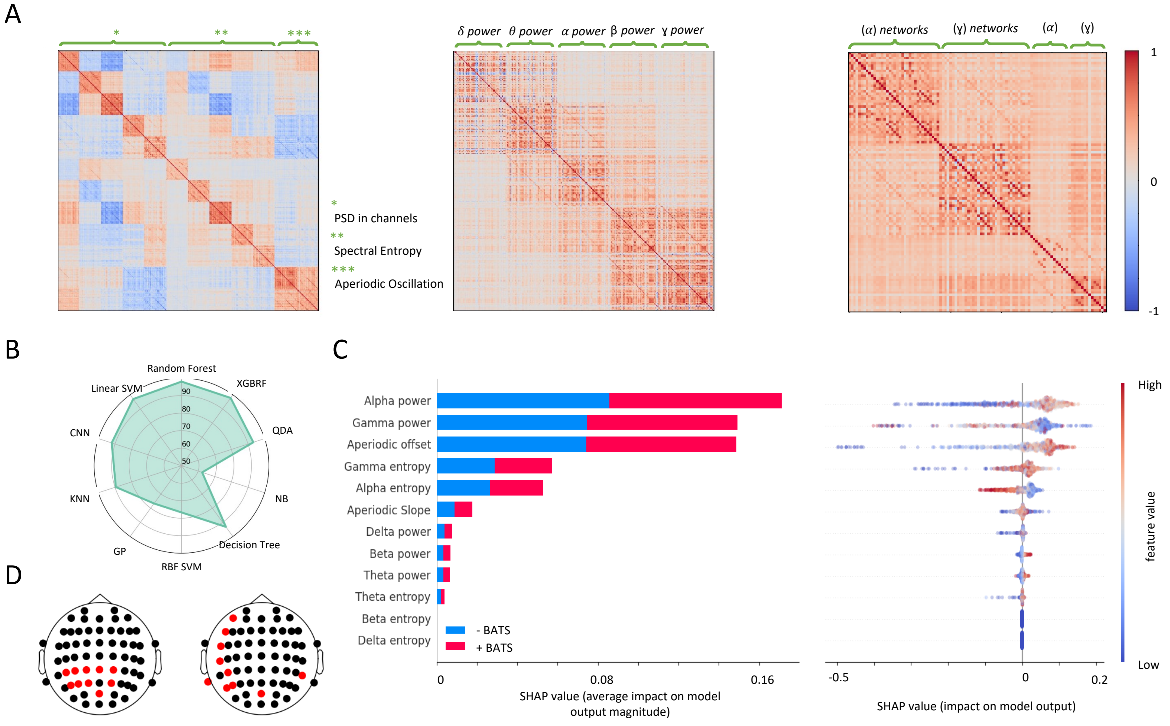

Figure S2. Analysis of validation dataset. A. Visual representation of the correlation matrix of the validation dataset, showing the relationships between various pairs of features including computed features in sensor space (left panel), source space (middle panel) and connectivity features (right panel). To reduce multicollinearity among features, values greater than 0.9 are combined. **B**. Accuracy values of ten distinct classifiers applied on the EEG epochs of validation dataset. **C**. Sensor space features are organized by decreasing importance for predicting two classes: +BATS and -BATS. In the left panel, the horizontal axis displays averaged SHAP values for each feature category, with higher values indicating greater influence on target prediction. Right panel consists of data points (epochs), representing feature categories, placed along the x-axis based on their SHAP values. Data point colors range from red to blue, reflecting feature values from high to low. **D**. Most contributing channels in classifying individuals with +BATS and -BATS are colored in red in both alpha (right panel) and gamma (left panel) frequency ranges.

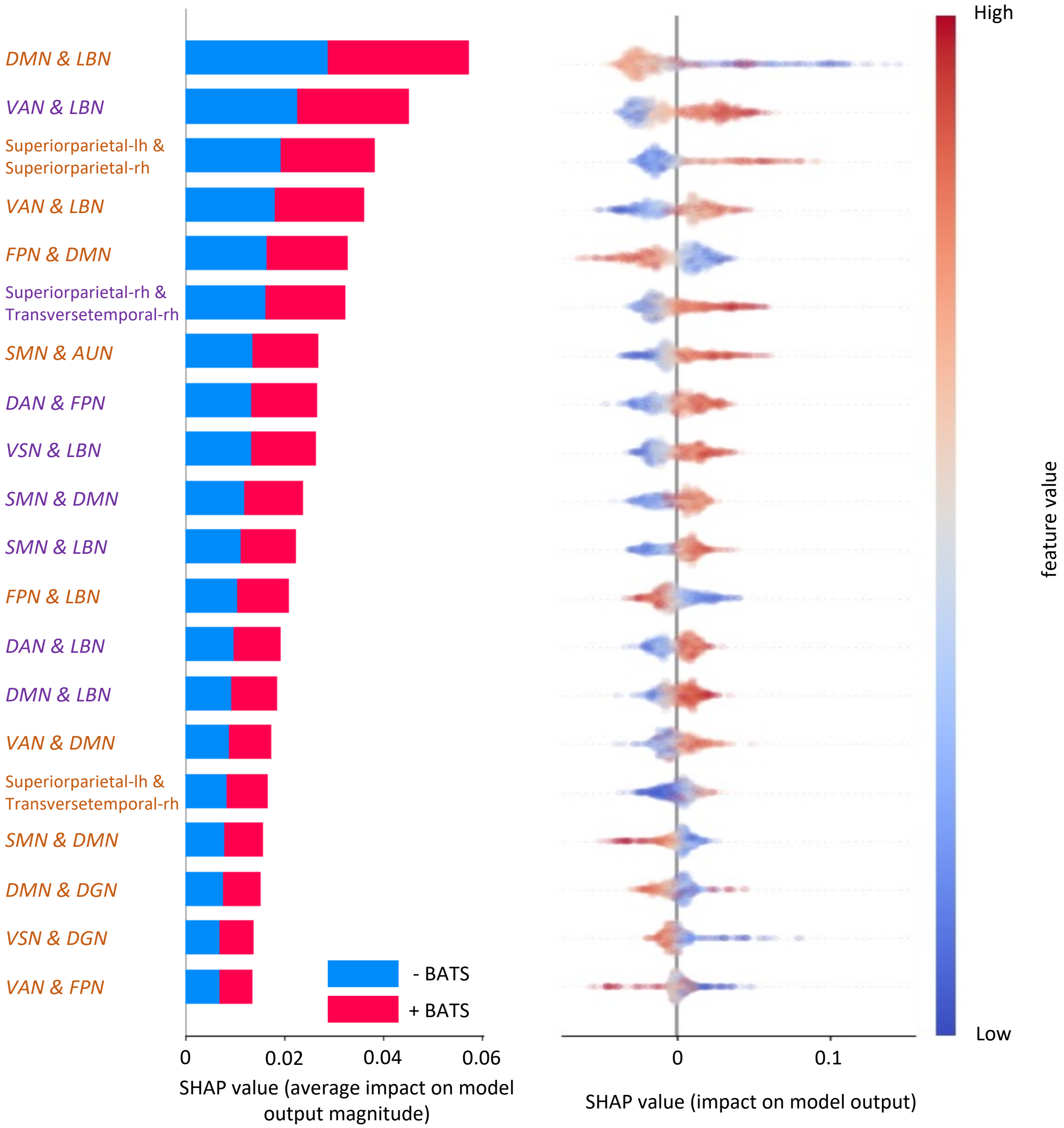

Figure S3. The list of most contributing connections in the validation dataset, sorted by their importance in the classification process. Each connection's directionality is also shown. The y-axis tick labels, colored in purple and light brown, correspond to connections in the alpha and gamma frequency ranges, respectively.

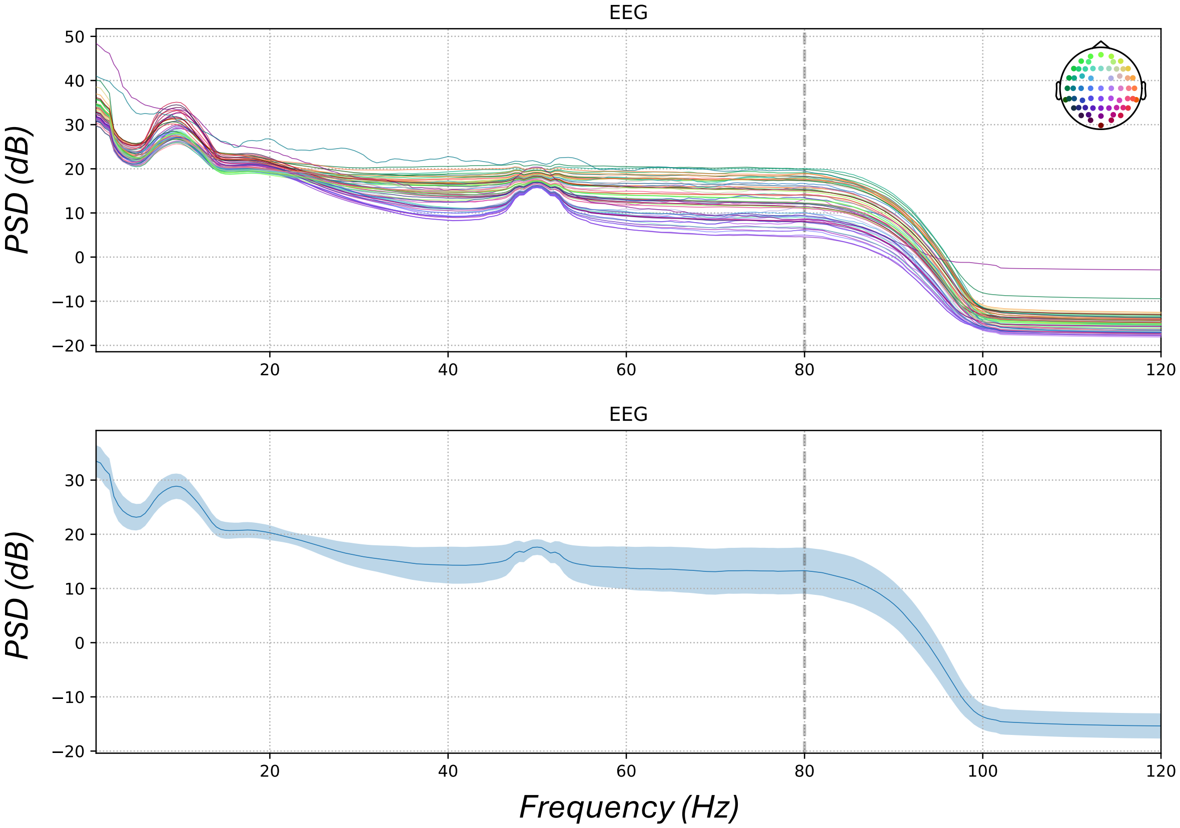

Figure S4. The power spectral density (PSD) of the main dataset, averaged across all subjects, is displayed for the 0–120 Hz frequency range. The top plot presents the PSD for individual channels, while the bottom plot features a blue ribbon illustrating the standard deviation of PSD across both subjects and channels. A dashed vertical line marks the high-cut frequency of the band-pass filter at 80 Hz.

References

1. Jurcak, V., Tsuzuki, D. & Dan, I. 10/20, 10/10, and 10/5 systems revisited: their validity as relative head-surface-based positioning systems. *Neuroimage* **34**, 1600–1611 (2007).
2. Oostenveld, R. & Praamstra, P. The five percent electrode system for high-resolution eeg and erp measurements. *Clin. neurophysiology* **112**, 713–719 (2001).
3. Li, A., Feitelberg, J., Saini, A. P., Höchenberger, R. & Scheltienne, M. Mne-icalabel: Automatically annotating ica components with iclabel in python. *J. Open Source Softw.* **7**, 4484 (2022).
4. Li, Z. *et al.* Objective recognition of tinnitus location using electroencephalography connectivity features. *Front. Neurosci.* **15**, 784721 (2022).
5. Li, J. *et al.* Exploring functional connectivity alterations in sudden sensorineural hearing loss: A multilevel analysis. *Brain Res.* **1824**, 148677 (2024).
6. Piarulli, A. *et al.* Tinnitus and distress: an electroencephalography classification study. *Brain communications* **5**, fcad018 (2023).
7. Gramfort, A. *et al.* Meg and eeg data analysis with mne-python. *Front. neuroscience* 267 (2013).
8. Donoghue, T. *et al.* Parameterizing neural power spectra into periodic and aperiodic components. *Nat. neuroscience* **23**, 1655–1665 (2020).
9. Li, A. *et al.* mne-connectivity (2022).
10. Pedregosa, F. *et al.* Scikit-learn: Machine learning in python. *J. machine Learn. research* **12**, 2825–2830 (2011).
11. Lundberg, S. M. & Lee, S.-I. A unified approach to interpreting model predictions. In Guyon, I. *et al.* (eds.) *Advances in Neural Information Processing Systems 30*, 4765–4774 (Curran Associates, Inc., 2017).
12. Buzsaki, G. *Rhythms of the Brain* (Oxford university press, 2006).
13. Percival, D. B. & Walden, A. T. *Spectral analysis for physical applications* (cambridge university press, 1993).
14. Slepian, D. Prolate spheroidal wave functions, fourier analysis, and uncertainty—v: The discrete case. *Bell Syst. Tech. J.* **57**, 1371–1430 (1978).
15. Desikan, R. S. *et al.* An automated labeling system for subdividing the human cerebral cortex on mri scans into gyral based regions of interest. *Neuroimage* **31**, 968–980 (2006).
16. Gini, C. W. Variability and mutability, contribution to the study of statistical distributions and relations. *Studi Econ. della R. Univ. de Cagliari* (1912).
